# Draft Genome Sequences of 12 Shiga Toxin-Producing Escherichia coli Strains Isolated from Dairy Cattle in Portugal

**DOI:** 10.1128/MRA.00790-20

**Published:** 2020-09-17

**Authors:** Gregor Fiedler, Andressa Ballem, Erik Brinks, Carina Almeida, Charles M. A. P. Franz, Hugo Oliveira

**Affiliations:** a Department of Microbiology and Biotechnology, Max Rubner-Institut, Federal Research Institute of Nutrition and Food, Kiel, Germany; b INIAV, IP—National Institute for Agrarian and Veterinary Research, Vairão, Vila do Conde, Portugal; c Centre of Biological Engineering, University of Minho, Braga, Portugal; University of Maryland School of Medicine

## Abstract

Shiga toxin-producing Escherichia coli (STEC) is a foodborne pathogen transmitted from animals to humans through contaminated food. Cattle are the main reservoir of STEC, but their genetic diversity is still poorly characterized, especially regarding strains isolated in Portugal. We therefore present the draft genomic sequences of 12 STEC strains isolated from cattle in the north of Portugal.

## ANNOUNCEMENT

Shiga toxin-producing Escherichia coli (STEC) is able to produce Shiga toxin as its major virulence factor, which is encoded by prophage regions (1). These prophages act as mobile elements and are therefore a driving force in the dissemination of Shiga toxin genes among STEC, other E. coli pathotypes, and members of *Enterobacterales* (2). STEC uses the gut of ruminant animals as a natural reservoir and is generally transmitted to humans through the consumption of contaminated foods. From the 500 STEC serotypes known to infect humans, only a few serotypes are responsible for the majority of foodborne cases (3).

In Portugal, the genetic diversity of STEC strains present in cattle has never been evaluated. To increase our knowledge of STEC population epidemiology, the repertoire of STEC genome sequences, and ecology, whole-genome sequencing was performed on 12 strains isolated from dairy cows on a specific farm located in Guilhabreu, Vila do Conde, Portugal.

The genome characteristics and virulence profiles are presented in Table 1. STEC strains were isolated from feces by enrichment with modified tryptone soy broth medium (supplemented with 20 mg liter^−1^ of novobiocin) and finally cultivated on both MacConkey sorbitol and tryptone bile X-glucuronide agar. Next, typical E. coli colonies were screened for the presence of *stx* genes by multiplex quantitative PCR (qPCR) according to International Organization for Standardization technical specification 13136:2012. Apart from strains E7N6P4C8A, E7N6P4C8C, and E7N6P4C8F, which are clones isolated from the same animal, the remaining strains originated from different cows/heifers. Strains were grown overnight in brain heart infusion broth at 37°C, and genomic DNA was extracted using the ZR fungal/bacterial DNA miniprep kit (Zymo Research, Freiburg, Germany). The DNA was quantified using a Qubit 3 fluorometer (Invitrogen, Darmstadt, Germany). The sequencing library was prepared with a TruSeq Nano DNA prep kit (Illumina, San Diego, CA, USA) and run on an Illumina MiSeq instrument with 2 × 251-bp paired ends using the MiSeq reagent kit v2 (Illumina). The raw data were trimmed with BBDuk (trimming adapters and low-quality reads and discarding reads shorted than 50 bp) (4), normalized (target coverage, 50×; minimum depth, 6×), and assembled with the Geneious Prime 2020 (Biomatters Ltd., New Zealand) *de novo* assembler with the medium/low-sensitivity settings (5). Read quality and assembly quality were assessed with FastQC v0.11.5 (6) and the Geneious Prime assembler, respectively. Contigs shorter than 500 bp or with coverage less than 8× were removed. The assembly *N*_50_ values ranged from 111.973 to 299.997 kb. The assembled contigs were screened using VirulenceFinder (7), SerotypeFinder (8), and PHASTER (9) with default parameters for *in silico* typing of *stx* genes and identification of prophages. PHASTER retrieves and classifies a given prophage as incomplete, questionable, or intact on the basis of its genome completeness or potential viability. Draft genomes were annotated using the NCBI Prokaryotic Genome Annotation Pipeline v4.12 (10). The genomic data show a diversity of STEC serotypes and virulence genes, with nine different serotypes detected on a single farm.

**TABLE 1:** Sequencing features of STEC strains

Strain	SRA accession no.	GenBank accession no.	*N*_50_ (bp)	No. of contigs	No. of raw reads	Genome coverage (×)	Genome size (bp)	No. of CDS^*a*^	GC content (%)	Serotype	No. of prophages	Shiga toxin gene type(s)	Other virulence genes	gMLST^*b*^
E7V3P1C1	SRR12187013	JACBWP000000000	113,570	82	3,095,354	62	5,459,339	5,376	49.5	O1:H10	14	*stx*_1_	*astA*, *ehxA*, *espI*, *hra*, *katP*, *lpfA*, *sta1*, *terC*, *traT*	1258
E7V4P1C10	SRR12187014	JACBWO000000000	147,484	78	3,120,412	63	5,458,642	5,362	49.4	O1:H10	12	*stx*_1_	*astA*, *ehxA*, *espI*, *espP*, *gad*, *hra*, *katP*, *lpfA*, *sta1*, *terC*, *traT*	1258
E7V12P1C4G	SRR12187005	JACBWN000000000	126,836	105	3,366,256	64	5,352,924	5,449	49.9	O182:H25	25	*stx*_1_	*astA*, *eae*, *ehxA*, *espA*, *espF*, *espJ*, *espP*, *etpD*, *gad*, *iss*, *lpfA*, *nleA*, *nleB*, *ompT*, *tccP*, *terC*, *tir*, *traT*	300
E7N3P7C8A	SRR12187006	JACBWM000000000	130,890	121	2,751,882	62	5,547,926	5,582	51.1	O150:H2	25	*stx*_1_, *stx*_2_	*astA*, *eae*, *ehxA*, *espA*, *espF*, *espI*, *espJ*, *espP*, *gad*, *iha*, *iucC*, *iutA*, *lpfA*, *nleA*, *nleB*, *ompT*, *tccP*, *terC*, *tir*, *traT*	306
E7N6P4C8A	SRR12187007	JACBWL000000000	210,932	76	3,759,806	60	5,302,937	5,130	51.3	O29:H12	14	*stx*_1_, *stx*_2_	*cea*, *celb*, *cia*, *ehxA*, *epeA*, *gad*, *hra*, *iha*, *lpfA*, *mchB*, *mchC*, *mchF*, *ompT*, *terC*	155
E7N6P4C8C	SRR12187008	JACBWK000000000	242,239	87	2,707,852	55	5,306,582	5,133	51.0	O29:H12	15	*stx*_1_, *stx*_2_	*cea*, *celb*, *cia*, *ehxA*, *epeA*, *gad*, *hra*, *iha*, *lpfA*, *mchB*, *mchC*, *mchF*, *ompT*, *terC*, *terC*	155
E7N6P4C8F	SRR12187009	JACBWJ000000000	160,056	79	3,083,908	59	5,302,542	5,126	51.0	O29:H12	15	*stx*_1_, *stx*_2_	*cea*, *celb*, *cia*, *ehxA*, *epeA*, *gad*, *hra*, *iha*, *lpfA*, *mchB*, *mchC*, *mchF*, *ompT*, *terC*	155
E7N8P4C1	SRR12187010	JACBWI000000000	238,307	83	2,368,706	51	5,271,944	5,113	51.4	O113:H21	13	*stx*_2_	*cdtB*, *cea*, *celb*, *cia*, *cib*, *ehxA*, *epeA*, *espP*, *gad*, *hra*, *iha*, *iss*, *lpfA*, *ompT*, *subA*, *terC*, *traT*	223
E7N12P2C4	SRR12187011	JACBWH000000000	127,918	144	2,996,158	52	5,811,737	5,937	51.1	O26:H11	22	*stx*_1_	*astA*, *celb*, *cif*, *eae*, *efa1*, *ehxA*, *espA*, *espB*, *espF*, *espJ*, *espP*, *fyuA*, *gad*, *iha*, *irp2*, *iss*, *iucC*, *iutA*, *katP*, *lpfA*, *nleA*, *nleB*, *nleC*, *ompT*, *tccP*, *terC*, *tir*, *toxB*, *traT*	21
E7N15P4C10	SRR12187012	JACBWG000000000	155,331	72	1,999,722	53	5,182,995	4,968	51.2	O116:H21	11	*stx*_2_	*cba*, *cdtB*, *cea*, *celb*, *cia*, *ehxA*, *epeA*, *espP*, *gad*, *hra*, *iha*, *lpfA*, *ompT*, *subA*, *terC*, *traT*	Unknown
E7N16P4C9	SRR12187015	JACBWF000000000	142,475	87	2,079,114	48	5,328,386	5,173	51.1	O29:H12	17	*stx*_1_, *stx*_2_	*cea*, *celb*, *cia*, *ehxA*, *epeA*, *gad*, *hra*, *iha*, *lpfA*, *mchB*, *mchC*, *mchF*, *ompT*, *terC*	155
E7N18P5C8G	SRR12187016	JACBWE000000000	299,977	44	2,479,862	54	5,084,573	4,977	51.9	ONT:H28	10	*stx*_1_, *stx*_2_	*cea*, *cia*, *cib*, *cvaC*, *ehxA*, *epeA*, *espP*, *gad*, *hra*, *iha*, *iss*, *lpfA*, *ompT*, *subA*, *terC*, *traT*	156

a CDS, DNA coding sequences.

b gMLST, genomic multilocus sequence typing.

### Data availability.

The assembled genome and sequencing reads were deposited under the BioProject accession number PRJNA643688. The GenBank and Sequence Read Archive (SRA) accession numbers are presented in Table 1.
